# Intussusception caused by heterotopic gastric mucosa in small intestine: a case report

**DOI:** 10.1186/s13256-017-1425-x

**Published:** 2017-09-12

**Authors:** Priyanka Anand, Sompal Singh, Namrata Sarin

**Affiliations:** Department of Pathology, NDMC and Hindu Rao Hospital, Malka Ganj, New Delhi, 110007 India

**Keywords:** Heterotopic, Gastric metaplasia, Intussusception, Intestinal

## Abstract

**Background:**

Intestinal intussusception is the most frequent cause of small bowel obstruction in children between the ages of 2 months and 5 years and often remains idiopathic in etiology, even after surgery. On microscopic examination, in intussusception normal mucosa is noted but in a few cases heterotopic tissue can be seen. Heterotopic gastric mucosa in the small intestine is extremely rare except for its occurrence in remnants of Meckel’s diverticulum. In view of the rarity of this condition, we report a case of ectopic gastric mucosa in the small intestine that was not associated with remnants of vitelline duct.

**Case presentation:**

A 6-year-old boy of Indo-Aryan ethnicity from India presented with episodes of acute abdominal pain and distension with vomiting and non-passage of stools. On ultrasonography intussusception was suspected. A laparotomy was done and the ileal segment (tip of intussusception) was sent for histopathological examination. On histopathology, sections from the tip of intussusception showed extensive gastric metaplasia of the mucosa.

**Conclusions:**

A definitive diagnosis of heterotopic gastric mucosa is established by histopathological examination and it is important to differentiate heterotopia, which is a developmental anomaly, from metaplasia, which is an acquired condition. Heterotopic gastric mucosa is usually clinically silent and surgical intervention can be considered in patients with complications such as gastrointestinal hemorrhage and intestinal obstruction.

## Background

Intestinal intussusception is the most frequent cause of small bowel obstruction in children between the ages of 2 months and 5 years; it often remains idiopathic in etiology, even after surgery. According to a study, approximately 75% of children with intussusception caused by intestinal obstruction were in their first year of life, which is in concordance with other studies where the commonest age group affected was below 1 year [1, 2]. However, after 2 years of age, an increasing number of intussusceptions are due to lesions of the small intestine, namely Meckel’s diverticulum, Henoch–Schönlein purpura, and Peutz–Jeghers polyps [3].

On microscopic examination, in intussusception normal mucosa is noted but in a few cases heterotopic tissue can be seen. In view of the rarity of this condition, we report a case of a 6-year-old boy with heterotopic gastric mucosa (HGM) in the small bowel.

## Case presentation

A 6-year-old boy of Indo-Aryan ethnicity from the northern part of India presented with episodes of acute abdominal pain and abdominal distension with vomiting and non-passage of stools. He denied a history of taking any medications in the past and his family history was not contributory. A physical examination was normal except for slight pallor. There was no hepatosplenomegaly. His complete blood counts, erythrocyte sedimentation rate (ESR), and serum electrolytes were within normal limits. On ultrasonography intussusception was suspected along with enlarged multiple mesenteric lymph nodes. At laparotomy, small bowel intussusception was found which was irreducible so a resection anastomosis was performed and the ileal segment (tip of intussusception) was sent for histopathological examination.

**Fig. 1:**
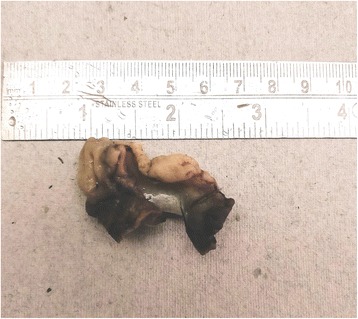
Gross photograph showing small intestinal segment measuring 2.5 × 1.5 cm

**Fig. 2:**
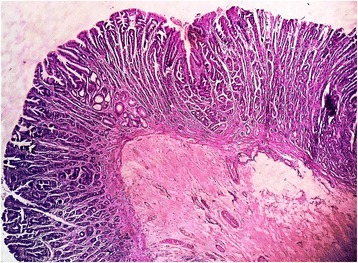
Photomicrograph showing gastric metaplasia in the overlying mucosa (×4, hematoxylin and eosin stain)

**Fig 3:**
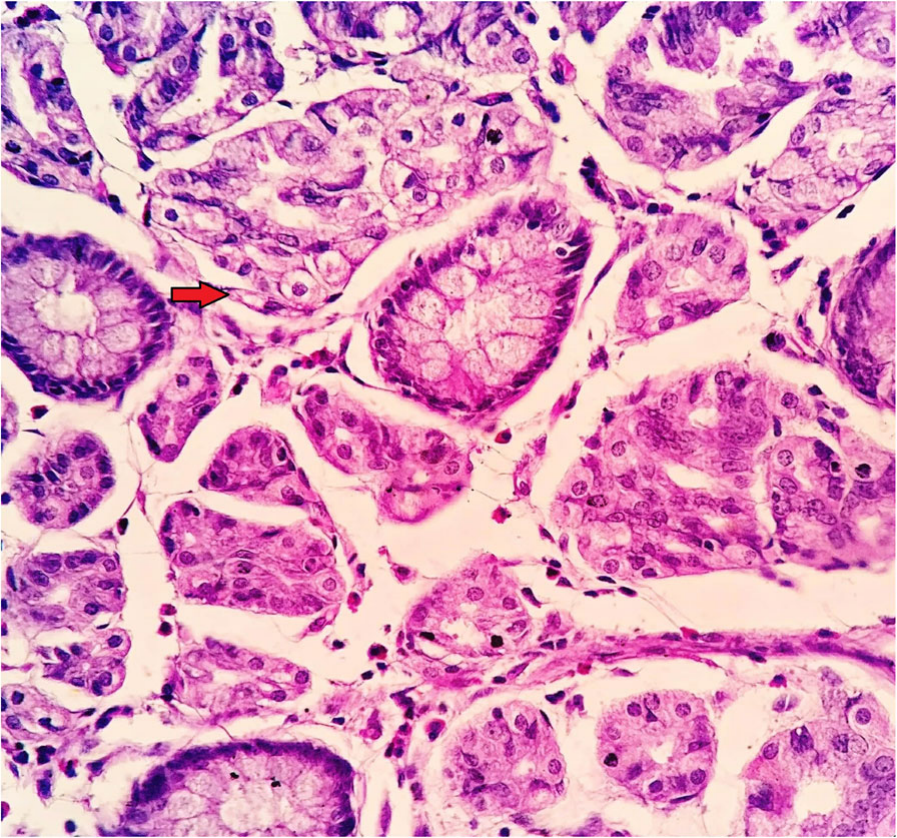
Photomicrograph showing the presence of parietal cells as shown by an *arrow* (×40, hematoxylin and eosin stain)

We received a part of the ileal segment measuring 3 × 1.5 cm; the external and cut surfaces were unremarkable (Fig. 1). On histopathology, sections from the tip of intussusception showed extensive gastric metaplasia of the mucosa with long coiled branching glands containing both abundant chief and parietal cells (Figs. 2 and 3). Following an uneventful postoperative outcome, he recovered satisfactorily and is currently under follow up.

## Discussion

Aberrant gastric gland is the term used where gastric glands are found in an organ other than the stomach. HGM of the intestinal tract is an occasional incidental gross or microscopic finding at surgery or autopsy [4]. HGM was described by Schmidt as early as 1805 [5]. Poindecker reported the first case of HGM in 1912 [6].

It is classified as either acquired metaplasia as in Barrett’s esophagus or a true gastric heterotopia of congenital origin as in Meckel’s diverticulum. The biggest diagnostic dilemma is of a simple true congenital gastric heterotopia being misdiagnosed as a gastric metaplasia [4].

HGM may be found anywhere in the gastrointestinal tract from the tongue to the rectum [7]. It is commonly found in congenital abnormalities such as Meckel’s diverticulum and gastrointestinal duplications [8]. However, it has been reported throughout the entire alimentary tract from the oral cavity to the anus, in the airways, umbilicus, urinary bladder, and even in the scrotum. The incidence of HGM in the esophagus varies widely from 0.1 to 13.8% and that of HGM in the duodenum varies from 0.5 to 8.9% [9]. Gastric heterotopias are mostly seen in the duodenum but very rarely in the small intestine as was found in our case [10].

Clinical presentation varies and depends on the size and location of HGM. It usually presents as an inlet patch in the proximal esophagus, as polypoid masses in the rectum, or as nodular tumors in the duodenum [11, 12]. Most of the patients are symptomatic and present with intermittent abdominal pain secondary to intestinal obstruction, chronic intussusception, perforated ulceration, or intestinal bleeding [12–15]. It may serve as the leading cause for the development of intussusception. The cause of the ulcer is due to acid pepsin secreted by the ectopic gastric mucosa [4]. It can also cause failure to thrive because of the chronic abdominal pain associated with recurrent episodes of vomiting and diarrhea [16]. Radioisotope scan is the investigation of choice to detect bleeding ectopic gastric mucosa.

Depending on the location, three mechanisms have been suggested for the development of HGM. One possibility is that during the developmental descent of the stomach, remnant tissue could have remained in the distal esophageal portion of the gut. However, although this mechanism could explain gastric heterotopia of the esophagus, it cannot explain gastric heterotopia in more distal intestinal tracts [17].

It has also been said that the condition may be acquired and is the result of an abnormal regenerative process following destruction of the normal intestinal mucosa. Acquired HGM is common in the jejunum and ileum in areas of mucosal regeneration accompanying inflammatory lesions such as regional enteritis [18]. However, this theory is not supported by any of the descriptions of gastric heterotopia following destruction of the gastrointestinal mucosa due to gastroenteritis or other inflammatory conditions [19].

The third and most possible hypothesis is that HGM is the result of abnormal differentiation of local tissue. Pluripotent primitive endoderm stem cells have the ability to differentiate into cell types of the gastrointestinal epithelium. An error in differentiation could lead to the gastric mucosa being present anywhere throughout the gastrointestinal tract [17, 19].

It is important to differentiate heterotopias from metaplasias. Metaplasia is a change of one type of full developed tissue to another differentiated tissue usually due to sustained inflammation and its complications. Heterotopias imply a developmental anomaly, whereas metaplasias imply an acquired condition. The common sites of gastric metaplasia occurring as a protective response to the injurious action of gastric acid are the lower esophagus and duodenal bulb.

In a case of acquired HGM, gastric mucosa consists mainly of mucus-secreting cells with the appearance of gastric pyloric glands. Parietal and chief cells are absent or, if present, are sparse. However, HGM consists of full mucosal thickness of structured gastric fundic mucosa consisting mainly of chief and parietal cells; the abnormality is considered developmental or congenital in origin as was seen in our case [20]. Thus, whereas heterotopias form a perfect mucosal island, metaplasias are of partial thickness and intermingle with the native tissue.

## Conclusions

Definitive diagnosis of HGM is established by histopathological examination and it is important to differentiate heterotopia, which is a developmental anomaly, from metaplasia, which is an acquired condition. It is usually clinically silent and does not require treatment. However, surgical intervention can be considered in patients with complications such as gastrointestinal hemorrhage and intestinal obstruction [21].

## Abbreviations

ESR: Erythrocyte sedimentation rate
HGM: Heterotopic gastric mucosa
